# Broad-spectrum anti-tumor and anti-metastatic DNA vaccine based on p62-encoding vector

**DOI:** 10.18632/oncotarget.1397

**Published:** 2013-10-02

**Authors:** Franco Venanzi, Victor Shifrin, Michael Y. Sherman, Vladimir Gabai, Oleg Kiselev, Andrey Komissarov, Mikhail Grudinin, Maria Shartukova, Ekaterina A. Romanovskaya-Romanko, Yuri Kudryavets, Natalya Bezdenezhnykh, Oleksandra Lykhova, Nadiia Semesyuk, Antonio Concetti, Anatoly Tsyb, Marina Filimonova, Victoria Makarchuk, Raisa Yakubovsky, Andrey Chursov, Vita Shcherbinina, Alexander Shneider

**Affiliations:** ^1^ Laboratory of Translational Biology, Department of Biology MCA, University of Camerino, 62032, Italy; ^2^ CureLab Oncology, Inc, Needham, MA, 02492, USA; ^3^ Dept Biochem, Boston University School of Medicine, Boston MA, 02118, USA; ^4^ Research Institute of Influenza, St-Petersburg, 197376, Russia; ^5^ R.E.Kavetsky Institute of Experimental Pathology, Oncology and Radiobiology of NAS of Ukraine, Kiev, 03022, Ukraine; ^6^ Medical Radiology Reseach Center, Obninsk, 249036, Russia; ^7^ Gertzen Research Oncology Institute, Moscow, 125284, Russia

## Abstract

Autophagy plays an important role in neoplastic transformation of cells and in resistance of cancer cells to radio- and chemotherapy. p62 (SQSTM1) is a key component of autophagic machinery which is also involved in signal transduction. Although recent empirical observations demonstrated that p62 is overexpressed in variety of human tumors, a mechanism of p62 overexpression is not known. Here we report that the transformation of normal human mammary epithelial cells with diverse oncogenes (RAS, PIK3CA and Her2) causes marked accumulation of p62. Based on this result, we hypothesized that p62 may be a feasible candidate to be an anti-cancer DNA vaccine. Here we performed a preclinical study of a novel DNA vaccine encoding p62. Intramuscularly administered p62-encoding plasmid induced anti-p62 antibodies and exhibited strong antitumor activity in four models of allogeneic mouse tumors – B16 melanoma, Lewis lung carcinoma (LLC), S37 sarcoma, and Ca755 breast carcinoma. In mice challenged with Ca755 cells, p62 treatment had dual effect: inhibited tumor growth in some mice and prolonged life in those mice which developed tumor size similar to control. P62-encoding plasmid has demonstrated its potency both as a preventive and therapeutic vaccine. Importantly, p62 vaccination drastically suppressed metastasis formation: in B16 melanoma where tumor cells where injected intravenously, and in LLC and S37 sarcoma with spontaneous metastasis. Overall, we conclude that a p62-encoding vector(s) constitute(s) a novel, effective broad-spectrum antitumor and anti-metastatic vaccine feasible for further development and clinical trials.

## INTRODUCTION

Immunotherapy is a rapidly developing approach toward cancer therapy. Several immune modulators such as anti-PD1 or anti-CTLA-4 antibody have been successfully used in clinics and in the FDA approval process [1]. DNA vaccines constitute a promising segment within cancer immunotherapy. The first anti-melanoma DNA vaccine (Oncept) is approved already for veterinary application [2]. DNA vaccines may possess numerous advantages as compared to traditional anticancer drugs including, but not limited to, minimal toxicity, immunological memory, and the fact that they could be used both for therapeutic and preventive purposes. Anti-cancer DNA vaccines have to be developed against antigens exposed on the cell surface as well as intracellular oncoproteins (e.g. survivin, WT-1, PRL-3 and others [3-5]).

Despite great promise, DNA vaccines generally elicit insufficient immune response against tumors. While being strongly immunogenic in mice, many DNA vaccines failed to elicit adequate immune response in bigger animals and humans. Multiple approaches have been tested providing a basis for hope that this bottleneck can be resolved, including advanced methods of delivery (e.g. electroporation or needle-free transdermal delivery [6, 7]), novel adjuvants and methods of antigen modifications specifically designed for DNA vaccines [8, 9], heterologous prime-boost vaccination regimens [10-12], combination with immunomodulators like anti-PD1 or anti-CTLA-4 antibody, or antagonists of A2A receptor [13]. Despite the improving response to antigens, these approaches only extend life expectancy of patients by several months, and eventually cancer relapses.

The major problem associated with anti-cancer DNA vaccines yet to be solved is that for the majority of tumor-associated antigens tested so far, vaccination applies selective pressure leading to the loss of the antigen (immunoediting), thereby resulting in the relapse of a tumor constituted of cells lacking the vaccine-encoded antigen. [14]. We argue that to avoid this problem one can utilize a protein crucial for survival of cancer cells as an antigen for the vaccination. In such a scenario, DNA vaccination would not be able to select cells lacking this protein. So far, most studies have not utilize antigens essential for cancer but dispensable for normal tissues. Our goal here was to find such an antigen, which can be used for potent anti-cancer vaccination with low chance of relapse. Here we describe a novel DNA vaccine based on p62 protein (sequestome 1) that is critical for cancer and dispensable for normal tissues. p62 performs two major functions in the cell – it is involved in autophagy [15, 16], and serves as a signaling hub for several signal transduction pathways such as NF-kB, p38, TRAF6, protein kinases etc [17]. Both functions of p62 are essential for tumor development. Indeed, the absence of p62 in the knockout mice completely prevented the emergence of cancer [18]. Furthermore, p62 was shown to be essential for growth and malignancy of several human tumor cell lines. Very importantly, from the perspective of vaccine development, the broad spectrum of human tumors demonstrate highly elevated p62 expression levels as compared to normal tissue [19-21]. Based on high intratumoral p62 level, together with the fact that p62 is indispensable for tumor formation and/or progression, we hypothesized that p62 may provide significant benefits as a potent antigen candidate for selective DNA vaccine, which a cancer would not be able to escape.

## RESULTS AND DISCUSSION

### p62 is Overexpressed in Various Cancers and Induced by Oncogenes

**Figure 1:**
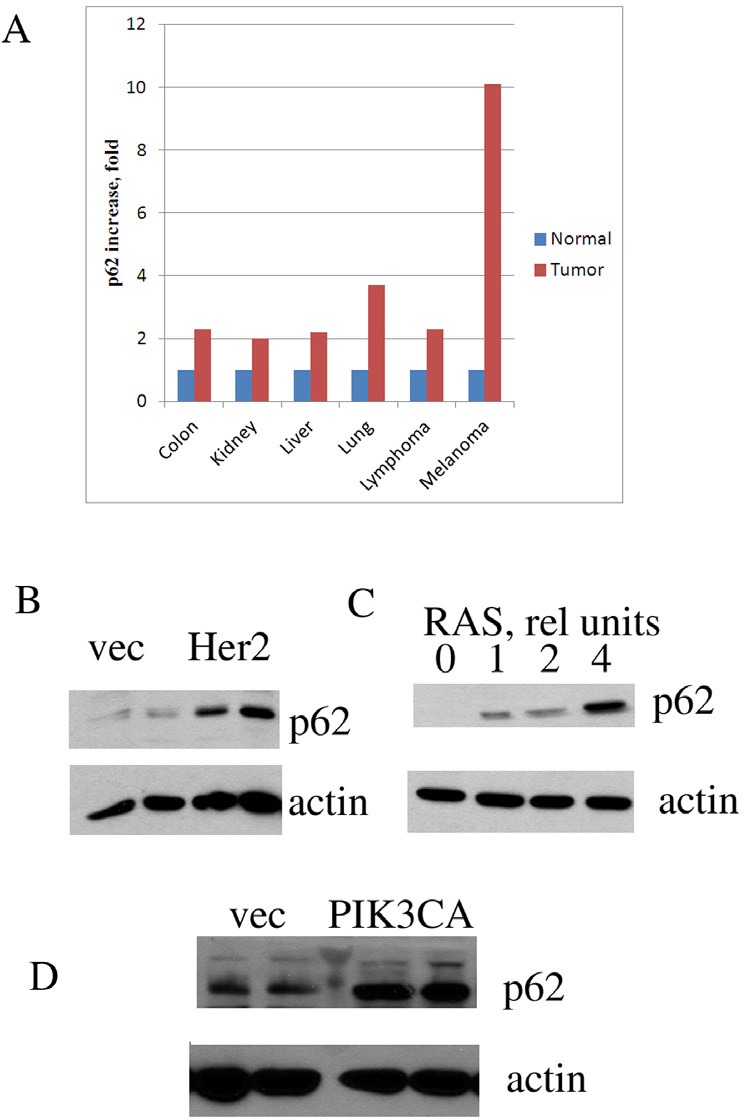
p62 is overexpressed in diverse human cancers and its expression is upregulated by oncogenes A. P62 mRNA expression in human cancers compared to corresponding normal tissues. The graph was derived from microarray data of Oncomine. B-D. Expression of oncogenes leads to p62 accumulation. MCF10A human mammary epithelial cells were transduced with retroviruses (B,C) or lentiviruses (D) expressing the corresponding oncogenes, and (after brief selection) levels of p62 were assessed by immunoblotting with anti-p62 antibody. Duplicates (separate infections) are shown in B and D, and effect of increased levels of RAS virus in C. Anti-actin antibody was used as a loading control.

First, we analyzed levels of p62 mRNAs in human tumors utilizing data from the Oncomine database. Results presented in Fig. 1A demonstrate that at least 6 types of cancers possessing elevated levels of p62 expression—from 2-fold in liver cancer to 10-fold in melanoma. Although mRNA level may not directly correlate with protein expression, our analysis was consistent with previously reported immunocytochemistry and immunoblotting data demonstrating p62 upregulation in breast and prostate cancers [19, 21]. Importantly, at least in case of breast cancer, p62 level increased during tumor progression [20].

Elevated levels of p62 in cancers may result from selection of clones with higher autophagy or stronger survival signaling (e.g. NF-kB), which may be necessary for survival in a tumor microenvironment. Alternatively, elevated expression of p62 may be a response to alteration of cancer-related signaling pathways in the process of cancer transformation or progression. To address these alternatives, we tested whether expression of common oncogenes in untransformed human breast epithelial cells MCF10A can induce p62 in culture. Under these conditions, cells are not under the stress of a tumor microenvironment, and therefore do not experience the selection pressure. MCF10A cells were infected with the retroviral vector encoding Ras oncogene followed by brief selection with puromycin. The rate of the retroviral infection was more than 80%, and therefore the selection was minimal. Of note, expression of Ras oncogene in MCF10A cells leads to their transformation [22]. Expression of Ras caused strong induction of p62. Furthermore, increasing the number of retroviral particles encoding Ras led to stronger p62 induction (Fig. 1C). Similarly, p62 induction occurred upon expression in MCF10A cells of other common oncogenes, including Her2 and PIK3CA (Fig. 1B,D). Thus, elevated levels of p62 in cancers are likely to be a direct consequence of the expression of oncogenes. Though it does not result from the selection of cells resistant to tumor microenvironment, p62 is critical for tumor survival, as mentioned above. Together these data indicate that elevated levels of p62 must be a stable feature of cancer cells.

### p62 Vaccine Demonstrates a Broad Spectrum Activity on Primary Tumors

**Figure 2:**
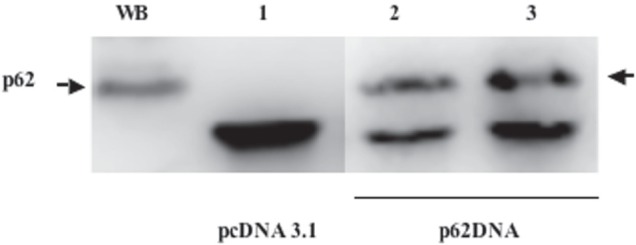
Appearance of p62 antibodies in mice serum after immunization with p62 vaccine WB - Western blotting of HeLa cells (positive control); 1) Immunoprecipitation with serum from vector (pcDNA3.1) - immunized mice; 2) and 3) independent duplicates of immunoprecipitation with serum from p62-plasmid injected mice. Arrow indicates p62.

At the next step, we designed a p62-based prototype DNA vaccine by inserting a p62 gene into pcDNA3.1 plasmid vector. Intramuscular administration of this vaccine evokes antibody response, i.e. appearance of antibody to p62 in mouse serum (Fig. 2). Since human melanoma and lung cancer demonstrated the highest levels of p62 overexpression as compared to normal tissues (see above Fig. 1A), we first selected Lewis lung carcinoma (LLC) and B16 melanoma mouse models for testing anti-cancer potency of our vaccine. These models are well-established, fast-growing and lethal to animals within one month after the challenge. Importantly, the tumor strains we utilized develop both primary tumors as well as metastases, so their effect on both components of cancer pathogenesis can be assessed.

**Figure 3:**
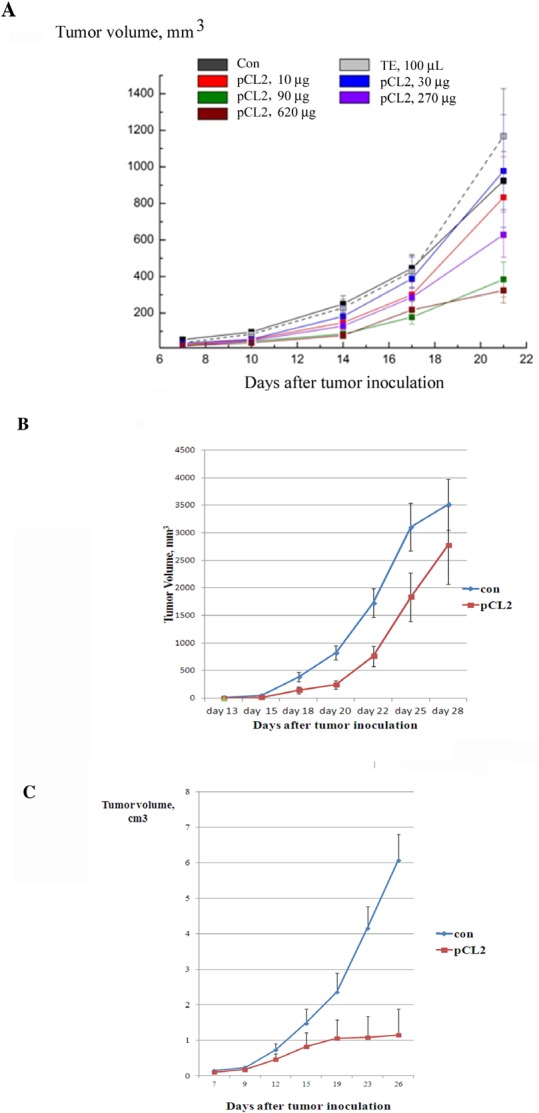
Anti-cancer activity of p62 vaccine in mouse solid tumors A. Dose-effect of p62 on LLC. Mice were treated with p62-encoding plasmid (pCL2) 14 and 7 days prior to tumor inoculation, and then at days 1, 8 and 15 after the inoculation. Tumor volumes were monitored by caliper starting from day 10 after tumor inoculation. The effect is observed with p < 0.05 for doses more than 90 μg/mouse.B. P62 vaccine suppresses growth of B16 melanoma. Mice received injections of p62-encoding plasmid on days 1, 8 and 14 after tumor inoculation. Growth of tumors was monitored by caliper starting from day 15. The effect is observed with p < 0.05. C. P62 vaccine suppresses growth of S37 sarcoma. Mice were injected with p62-encoding plasmid 14 and 7 days prior to tumor inoculation, and then at days 1, 8, 15, 22 and 29 after the inoculation. Tumor growth was monitored by caliper starting from day 9. The effect is observed with p < 0.05 starting on day 15th.

First, we assessed the dose-response curve for p62 vaccine on LLC primary tumors. The vaccine was administered intramuscularly five times (2 weeks and 1 week prior to the tumor inoculation and 3 times after the tumor challenge at the days 1, 8 and 15). We assessed the dose-response curve for p62 vaccine. The vaccine was administrated intramuscularly at doses ranging from 10 to 600 ug/mouse). We observed a typical S-curve dose-response effect. There was no effect on primary LLC tumor growth at low doses (10 and 30 ug), but higher doses produced a very profound dose-dependent effect with a saturation point at dose 300 ug (Fig. 3A). Importantly, p62 vaccine did not demonstrate toxicity even at the highest dose (600 ug).

Having established the dose-effect curve, we applied p62 vaccine to the B16 melanoma model. In this model, we assessed therapeutic rather than prophylactic potential of the vaccine, which is more relevant to a clinical situation when therapies are administered after the formation of primary tumors. Since B16 melanoma grows in mice very rapidly, and it takes some time for the immune response to develop, we gave the first injection of p62 vaccine one day after inoculation of B16 melanoma cells, with two additional doses injected *im* on days 8 and 14 after tumor inoculation. Administered according to this schedule, p62 DNA vaccine demonstrated significant inhibition of growth of B16 melanoma primary tumors (Fig. 3B).

Next, we studied the effect of the vaccine on S37 mouse sarcoma. We observed a very strong antitumor effect when animals were treated with p62 vaccine 2 times before and 5 times after tumor inoculation with weekly intervals (Fig. 3C). Remarkably, 15 days following tumor inoculation, S37 sarcoma almost ceased to grow, whereas in control it continued to grow rapidly (Fig.3C).

**Figure 4:**
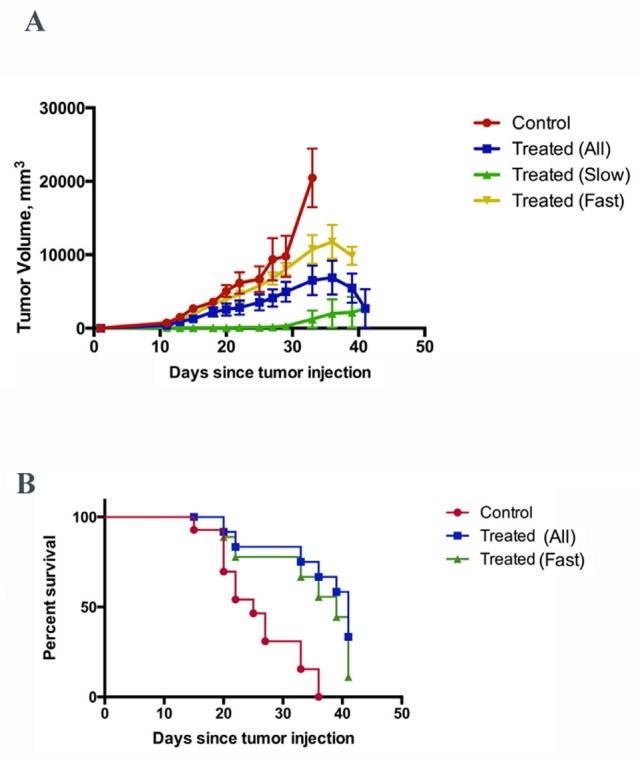
Effect of p62 vaccine on Ca755 breast carcinoma A. Growth curve of tumors in control (untreated) animals, and in animals treated with p62 vaccine (all tumors, slow growing tumors, and fast-growing tumors). See text for further explanation. B. Survival of animals in control, and animals treated with p62 vaccine (all and fast growing tumors). Control curve vs. treated curve - P= 0.0003; control curve vs. treated curve (fast) - P= 0.0042 (Logrank test).

Effect of p62 vaccine was also studied in Ca755 breast cancer model using the same vaccination scheme as with LLC. Ca755 is a rapidly growing tumor which lead to 50% mortality in control group within 25 days after the challenge (Fig. 4 A,B). In this model, we observed two different effects of p62 DNA vaccination on a primary tumor. The vaccine completely prevented or markedly delayed growth of tumors in some animals (Fig.4A, slow growing tumors), while in other animals tumors grew similar to control (Fig. 4A, fast growing tumors). Surprisingly, even in the subgroup with fast growing tumors, survival of mice was significantly increased leading to about 1.6-times longer overall survival (50% survival was 39 days, Fig. 4B). Thus, inhibition of tumor growth and delay in mortality caused by the tumors, which were similar in size to those in the control group, are two different mechanisms of action. Apparently, both mechanisms may be important for vaccine against breast cancer.

Overall, in all four mouse models of solid tumors, p62 vaccine demonstrated potent suppression of tumor growth.

### p62 Vaccine has a Strong Antimetastatic Effect

After establishing that p62 DNA vaccine demonstrates a broad spectrum effect against primary tumors, we asked whether our vaccine can limit the metastatic process, since usually it is metastases rather than the primary tumor that kills patients. To test the antimetastatic effect of p62 DNA vaccine, we used the same three models: subcutaneously administered LLC and S37, and intravenously injected B16 melanoma.

**Figure 5:**
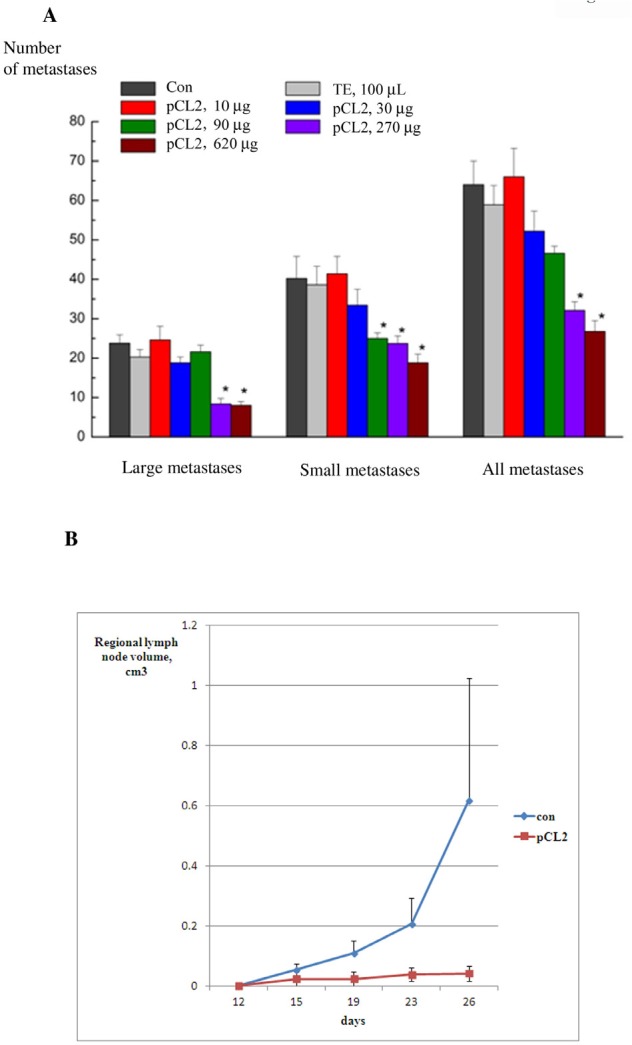
Anti-metastatic activity of p62 vaccine in LLC and sarcoma A. Dose-response of anti-metastatic activity of p62 in LLC. P62-encoding plasmid was administered in mice as in 3B. Mice were euthanized on day 21 and the number of metastases was counted and analyzed as described in Materials and Methods. We observe an effect with p<0.05 for doses 270 and 620 μg/mouse. B-D. Anti-metastatic activity of p62 vaccine in S37 sarcoma. Mice were treated with p62-encoding plasmid as in Fig. 3D, and the volume of regionary lymph nodes was monitored by caliper. P<0.05 on days 15-26.

LLC was inoculated and treated with increasing doses of p62 vaccine similar to the dose-effect curve described above for primary tumors. Mice were euthanized on day 21 after tumor inoculation and the number of small and large metastases in lungs was counted (see Materials and Methods for details). LLC generates 60 metastases per mouse on average (Fig. 5A). Low doses of p62, 10-90 ug/mouse do not affect the number of metastases; however, higher doses (between 270 and 620 ug/mouse), showed very significant antimetastatic effect, decreasing the number of metastases per lung (Fig. 5A). Similar to results with primary tumors, the saturation point was around 270 ug/mouse (Fig. 3A). Importantly, p62 vaccine decreased both the number of small and large metastases to a similar extent, indicating that the treatment prevents formation of new metastases instead of just inhibiting growth of already established metastases.

In the next model of metastases, antimetastatic activity of p62 vaccine was studied in S37 sarcoma, which produces metastases regionally in lymph nodes. As seen in Fig. 5B, application of the vaccine dramatically decreased the sizes (volumes) of metastases.

**Figure 6:**
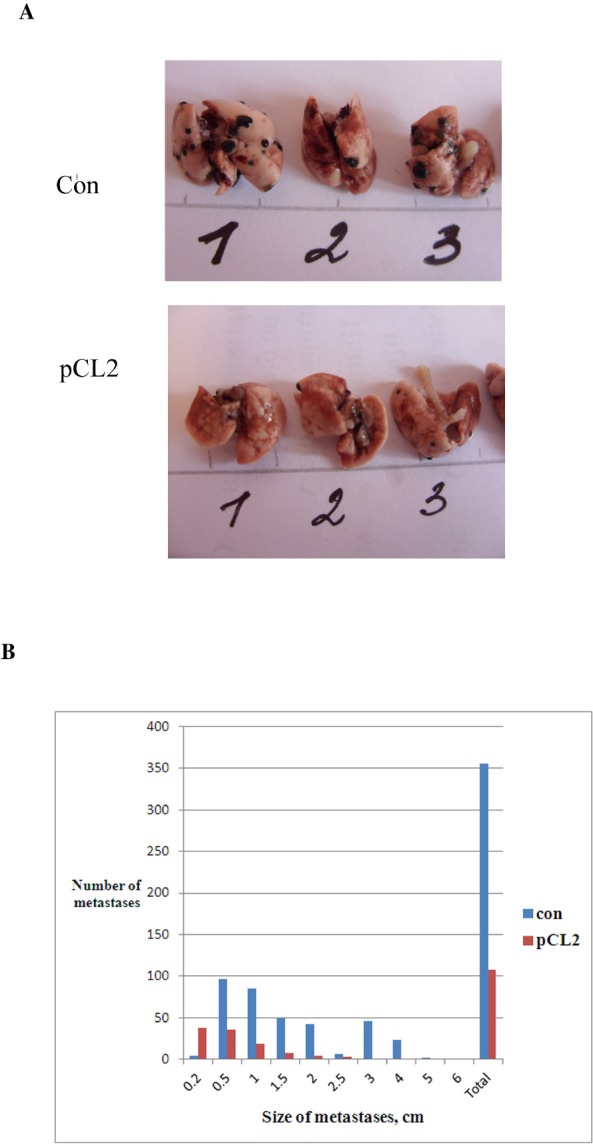
Anti-metastatic activity of p62 vaccine in B16 melanoma A, B. Mice were treated with p62-encoding plasmid as in Fig. 3B, but instead of inoculation of tumor cells s.c. they were injected i.v., and on day 28 a number and size of metastases were determined. Lung with metastases of melanoma (black) are shown in A. The effect is observed with p=0.036.

Finally, in the third model of metastases which we employed, B16 melanoma cells were injected in the tail vein of mice. This model allows clarifying whether p62 vaccine prevents formation of metastases when tumor cells are already in circulation, rather than their evasion from the primary tumor. This resembles the clinical situation when a primary tumor is eradicated (for instance, by surgery), but tumor cells are already in blood and are capable of forming metastases. Similar to the results presented above with B16 primary tumors, we used triple injection of p62 vaccine at days 1, 8 and 15 after inoculation of tumor cells. As seen in Fig.6, p62 vaccine caused significant decrease both the number of lung metastasis, and their size.

Overall, p62 DNA vaccine demonstrated strong anti-metastatic effect in all 3 models of metastatic process we have used.

We conclude that p62 may serve as an antigen for a broad-spectrum anti-cancer vaccine. Its unique feature is that p62 is essential for cancer cells, and thereby it limits the possibility of cancer relapse due to selection of clones that lack p62. Taken together, our data can be viewed as feasibility analysis and proof of concept for pre-clinical and clinical development of anti-cancer DNA vaccine based on p62.

## MATERIALS AND METHODS

### P62 Vaccine

Human p62 (SQSM1, isoform 1) was cloned in pcDNA3.1 vector. Plasmid DNA was purified using Endo-free Giga Kit (Qiagen). Quality of plasmid preparation for each experiment was assessed by DNA agarose electrophoresis and by sequencing.

### Cell Cultures, Treatments, and Reagents

MCF10A were from ATCC; MCF10A cells expressing PIK3CA were kindly provided by Dr. T. Waldmann. MCF10A cells were cultivated in Dulbecco's modified Eagle's medium-F12 medium supplemented with 5% horse serum, hydrocortisone (500 ng/ml), insulin (10 μg/ml), cholera toxin (100 ng/ml), epidermal growth factor ([20 ng/ml]), and penicillin/streptomicin. B16 melanoma cells (MM4 strain) (21) were grown in DMEM medium with 10% fetal bovine serum and penicillin/streptomycin. H-RAS V12 and control (Babe) retroviral vectors were kindly provided by S. Lowe, and Her2 retrovirus – by Dr. C Spangenberg. For production of retroviruses, we used 293T cells, which were cotransfected using Lipofectamine 2000 (Invitrogene) with plasmids expressing retroviral proteins Gag-Pol, G (vesicular stomatitis virus G protein pseudotype), and constructs of interest or enhanced green fluorescent protein. At 48 h after transfection, supernatants containing the retrovirus were collected and frozen at −70°C. Cells were infected with supernatant diluted 2× and 10 μg/ml Polybrene overnight and washed, then selection with puromycin (0.75 μg/ml) was started 48 h after infection. Retroviral vector expressing enhanced green fluorescent protein was used as a control for infection efficiency: usually 70 to 90% of cells were fluorescent 2 days after infection.

### Immunoblotting and Antibodies

Cells were washed twice with PBS, aspirated, and lysed as described [22]. Total protein concentration was measured in supernatants with the Bio-Rad protein assay, after which they were diluted with lysis buffer to achieve equal protein concentrations in all samples. The antibodies used were: anti- p62 – from BD Biosciences anti-β-actin from Sigma.

### Mice and Tumors

For experiments with Lewis Lung Carcinoma (LLC), B16 melanoma (strain MM-4 [23] and Ca755 breast carcinoma, we used C57Bl6 male mice at 7-8 weeks of age.

For experiments with S37 sarcoma, we used F1 hybrids of CBA × C57Bl/6 female mice.

P62 vaccine was injected i.m. in the volume of 100 ul and different concentrations (see Figures legends); empty vector or saline were used as controls and they did not have any effects in any experiments.

Tumor cells were injected subcutaneously in the leg in the amount of 7×10^5^ for LLC, 3×10^5^ for B16 melanoma, and 1×10^6^ per mouse for S37 sarcoma and Ca755 breast carcinoma. The size of tumor was measured every 2-3 days with a caliper, and the volume of tumor was calculated according to formula π/6 × L×W×H.

For measurement of spontaneous metastases in LLC mice, they were euthanized at day 21 after tumor inoculation, fixed with Bowen fixative and amounts of small and big lung metastases were calculated in each mouse. For measurement metastases in melanoma, 3×10^5^ cells per mouse were injected in lateral tail vein of mice and the amount of metastasis and their size was calculated in each mouse. In S37 sarcoma, regional metastases in lymph nodes adjacent to primary tumors were measured by caliper and their volume was calculated as above.

Statistical significance was assessed using Student t-test and Mann-Withey U-test using Statistica program.
